# Effects of hypoxia on survival, behavior, metabolism and cellular damage of Manila clam (*Ruditapes philippinarum*)

**DOI:** 10.1371/journal.pone.0215158

**Published:** 2019-04-18

**Authors:** Qiao Li, Song Sun, Fang Zhang, Minxiao Wang, Mengna Li

**Affiliations:** 1 CAS Key Laboratory of Marine Ecology and Environmental Sciences, Institute of Oceanology, Chinese Academy of Sciences, Qingdao, China; 2 University of Chinese Academy of Sciences, Beijing, China; 3 Laboratory for Marine Ecology and Environmental Science, Qingdao National Laboratory for Marine Science and Technology, Qingdao, China; 4 Center for Ocean Mega-Science, Chinese Academy of Sciences, Qingdao, China; 5 Jiaozhou Bay Marine Ecosystem Research Station, Institute of Oceanology, Chinese Academy of Sciences, Qingdao, China; Tanzania Fisheries Research Institute, UNITED REPUBLIC OF TANZANIA

## Abstract

The Manila clam *Ruditapes philippinarum* has become a common and dominant macrobenthic species in coastal areas of the northwestern Pacific and temperate waters of Europe; it is also a major cultured shellfish, with annual worldwide production exceeding 3.3 million tonnes. This species faces greater risk of exposure to hypoxia as eutrophication worsens throughout its coastal habitats; however, its tolerance to hypoxia remains unclear, and the toxicological indicators including LC_50_ and LT_50_ have not yet been assessed. Previous studies on the effects of hypoxia on marine benthos have focused largely on functional responses, such as metabolism and gene expression, leaving potential structural damage to the mitochondria or the cells unknown. In this study we assessed the effects of hypoxia on Manila clam in terms of survival, behavior, metabolism and cellular damage, using a newly designed automated hypoxia simulation device that features exceptional accuracy and good stability. The clams exhibited strong tolerance to hypoxia as the 20-day LC_50_ for dissolved oxygen (DO) was estimated to be 0.57 mg L^-1^, and the LT_50_ at 0.5 mg L^-1^ DO was 422 hours. Adaptations included fewer buried clams and a depressed metabolism, while the unexpected rise in the activities of key enzymes involved in glycolysis may indicate a diverse strategy of shellfish under hypoxia. Cellular damage was observed as collapse of the mitochondrial cristae and both cellular and mitochondrial vacuolization. This multi-level study complements and updates our knowledge of the effects of hypoxia on marine benthos, by improving our understanding of the potential for marine ecological transformation under hypoxic conditions and providing useful information for Manila clam farming.

## Introduction

As the eutrophication of coastal waters intensifies, both the extent and strength of oxygen depletion (hypoxia) increase [1,2]. Though certain mobile species can flee hypoxic zones, less-tolerant organisms with poor mobility often suffer mass mortalities under severe hypoxia [2–4], often leading to further harmful ecological changes, such as jellyfish blooms [5,6] and other forms of ecosystem degradation [7,8].

The commercially important Manila clam *Ruditapes philippinarum* has strong adaptability to a wide range of salinities and water temperatures; the species has a widespread natural distribution in coastal areas of the northwestern Pacific and has become established on the Atlantic coast of Europe. As a common and dominant species in many coastal regions, it functions as a key part of food webs and the biogeochemical cycle in these marine and brackish ecosystems [9–11]. Owing to its rapid growth and ample productivity, with more than 3.3 million tons produced globally per year, Manila clam supports aquaculture industries in China, Korea and Japan, and is among the top-25 aquatic species introduced in European countries like Spain, France, Italy and Ireland [12,13]. However, worsening hypoxia in shallow coastal waters and estuaries increasingly challenges the survival of Manila clam and mariculture industry development. In China, the severity of summer hypoxia has been reportedly rising in coastal areas of Shandong Peninsula, an important natural habitat and mariculture region for Manila clam. Dissolved oxygen (DO) in Laizhou Bay and Rushan Bay dropped below 2.0 mg L^-1^, the situation was worse in the Xiaoqing River estuary, with DO concentrations of less than 0.5 mg L^-1^ [14–16]. In Japan, mass mortalities of Manila clam caused by hypoxia have occurred many times during summer, bringing tremendous economic loss to the fishery [17,18].

The tolerance to hypoxia by marine benthos varies among taxa. Bivalves, such as Manila clam, are believed to be more tolerant than fishes and crustaceans. The mean LC_50_ for several common bivalves was estimated to be 1.42 ± 0.14 mg L^-1^, which is much lower than that estimated for some crustaceans (2.45 ± 0.14 mg L^-1^) [4]. In addition, different species of bivalves have different levels of tolerance; the LC_50_ for Atlantic surf clam *Spisula solidissima* was measured as 0.5 mg L^-1^, while for Baltic clam *Macoma balthica* it was 1.7 mg L^-1^ [19,20]. Previous studies have indicated that Manila clams are able to survive more than a week in severe oxygen-deficient conditions [17,18]. However, most experiments with this species have been conducted at a single extremely low DO level, often < 0.5 mg L^-1^, and there are no quantitative results for LC_50_ or LT_50_.

Animals have adopted a variety of strategies to survive hypoxia, from behavioral to metabolic. Typically, a buried bivalve will first ensure its oxygen supply by emerging from the sediment, to then enhance its respiratory rate and the oxygen-carrying capability of the blood. But if the hypoxia lasts for a relatively long time, a metabolic response may be crucial [21,22]. Farrer’s scallop *Chlamys farreri* and blue mussel *Mytilus edulis*, whose distribution overlaps with that of Manila clam, were found to have depressed their oxygen consumption rate (OCR), from 0.003 μg g^-1^ h^-1^ and 0.14 mg g^-1^ h^-1^ to 0.0005 μg g^-1^ h^-1^ and 0.05 mg g^-1^ h^-1^, respectively, when DO levels dropped from 6.0 mg L^-1^ to less than 2.0 mg L^-1^ [23]. The activities of enzymes, the driving force of metabolism, also react to hypoxic stress. In fishes, crustaceans and mollusks, antioxidant enzymes like superoxide dismutase and catalase are widely reported as being affected by hypoxia. As a natural response to protect an organism from physiological damage, these enzymes are usually immediately activated when DO level starts to drop; yet under long-term hypoxia their activities have a greater chance of becoming depressed, exposing the benthos to other potential damage via environmental stress [24–26]. However, respiratory enzyme activities, which are closely related to energy supply, have received little attention, especially in bivalves. Moreover, recent studies of Manila clam as well as other marine benthos have focused on functional responses, while structural responses at the cellular level are still unclear.

In the present research, long-term laboratory experiments were carried out to determine the effects of hypoxia on the survival, behavior, metabolism and cellular damage of *R*. *philippinarum*. A novel hypoxia simulation device, designed following the principle of hysteresis and with a few other innovations, was adopted for the first time, enabling us to precisely reduce the DO increment in the experimental design to as little as 0.2 mg L^-1^.

## Materials and methods

### Experimental animals and sediments

Manila clams used for the experiments were collected from Xidayang Cun Farm in Jiaozhou Bay (36°11'15"N, 120°16' 34"E) and the sampling was carried out by fishing boats using sucking pumps. The sampling was approved by the Xidayang Cun Council. All clams were transported to the laboratory within three hours. During transportation, the clams were held in storage boxes with seawater and with compressed air continuously pumped in. Before the experiments, all clams were brushed and kept in aquariums without sediment. Two-thirds of the water (salinity 30.0 ± 1.0) was replaced every two days, and *Chlorella vulgaris* (9.13 ± 2.48 mg L^-1^) was provided as food once a day. The water temperature was maintained at 22°C using heaters. Compressed air was pumped in for 30 minutes every 8 hours, and the DO concentration was closely monitored to ensure that it did not drop below 6.0 mg L^-1^. All aquariums were covered with 2-mm-thick black canvas to avoid light stimulus.

Natural sediments were collected from the intertidal zone at the same site where the clams were collected. During sampling, several surveying rods were inserted into the sediment to specify the depth so that only the top 12 ± 3 cm of sediment inhabited by Manila clams was collected. After transportation to the laboratory, the collected sediment was sieved through 2-mm mesh to remove other benthos and then placed in the experimental tanks to achieve a 12-cm sediment layer. The water above the sediment was 45 cm deep and there was no headspace.

Before being placed in the experimental tanks, the clams were carefully examined for their health and measured for weight and length. First, as a sign of health, the shells needed to be strictly intact and in a half-open state when there was no stimulus. Next, when the siphons were touched with a glass rod two times, the clam had to withdraw its siphons quickly. Among the clams satisfying these requirements, the ones with a similar weight and length were selected for the experiments (Table 1).

**Table 1:** Mean (± SD) shell length and wet weight of the *Ruditapes philippinarum* selected for the two hypoxia experiments.

Experiment	Total number	Shell length (mm)		Wet weight (g)	
Experiment 1	379	32.81 ± 1.44	No significant difference (analysis of variance, *p* < 0.01)	6.85 ± 0.49	No significant difference (analysis of variance, *p* < 0.01)
Experiment 2.1	380	32.50 ± 2.01		6.80 ± 0.52	
Experiment 2.2	384	31.98 ± 2.60		6.83 ± 0.44	
Experiment 2.3	381	32.35 ± 0.94		6.90 ± 0.59	
Experiment 2.4	384	32.68 ± 1.23		6.95 ± 0.57	

### A novel hypoxia simulation device

**Fig 1:**
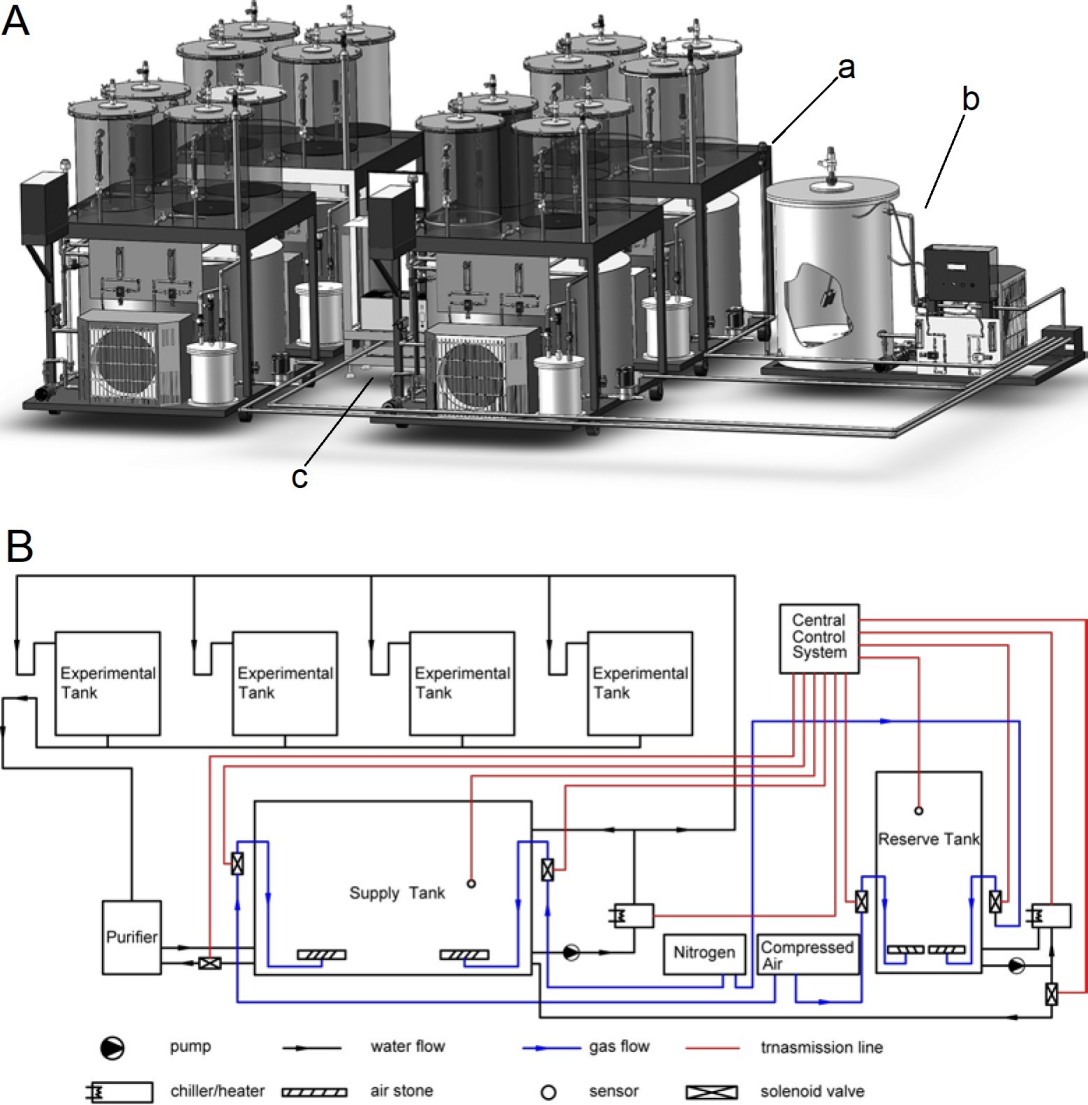
Design of the automated hypoxia simulation device. (A) The sketch showed the main structure of the device. The structure labeled with ‘a’ was the environmental simulation system; the structure labeled with ‘b’ was the water-exchange system; the structure labeled with ‘c’ was the central control system. (B) A logic diagram indicated how the device was running, including the water flow and gas flow.

To ensure more accurate and stable DO concentrations and to eliminate the disturbance introduced by bubbling in nitrogen or changing the tank water, and thereby to maintain good water quality during the long-term experiments, we developed an automated hypoxia simulation device which also featured a temperature-control function. The device consisted of four identical environmental simulation systems, a water-exchange system, and a central control system (Fig 1). For each environmental simulation system, a supply tank served as a chamber to allow adjustment of the DO concentration and water temperature; this was placed below four replicate experimental tanks. This design prevented the animals from being shocked by the environmental oscillations. The supply tank was equipped with both a chiller and a heater for temperature regulation, while DO adjustment was achieved by bubbling in nitrogen or compressed air under the control of solenoid valves. To measure DO and water temperature, sensors were installed inside the supply tank. Of these, the DO sensors were based on the principle of luminescence quenching, which offers a variety of advantages over traditional electro-chemical probes (e.g. no need for stirring; less potential for fouling; long-term stability without calibration; and faster response time) [27]. Once the system was functioning, seawater was pumped from the supply tank to the experimental tanks and then back again. Between them was a purifier to filter out the metabolic wastes produced by the clams. The environmental simulation system contained 1,160 L of seawater, with 800 L in the supply tank and 90 L in each experimental tank. The huge water capacity together with the recirculating arrangement ensured a fairly stable environment.

The water-exchange system featured a 900-L reserve tank which had a similar structure as the supply tank. When the system was activated, the DO concentration and temperature of the seawater in the reserve tank could be adjusted to the same levels as in the target supply tank. After that, a certain volume of seawater would be discharged from the target supply tank, and then an equal amount of seawater would be supplemented under the control of the water solenoid valves.

The central control system comprised a DO meter, a relay and a computer, which automatically actuated the compressed air or nitrogen solenoid valves for adjusting the DO level and also controlled the chiller or heater.

Since all sensors require time to output readings, the DO data received was often several minutes old. However, this design would lead to an excessive input of nitrogen or oxygen once the desired DO concentration had been reached. Worse, it would also lead to an excessive input of the gas since it took time for oxygen to spread evenly in the water. To effectively solve this problem, we employed the principle of hysteresis in designing the central control system. The hysteresis principle involves a control for switching logic; this took the time-delay described above into consideration, based on the relationship between the current DO reading, the actual DO value, and the DO spreading time. Thus, the actuation and closing of the solenoid valves could be performed before the DO reading exceeded the control range. In addition, the water exchange could also proceed automatically by the actuation of a corresponding water solenoid valve, after instructions were relayed.

The water supply and reserve tanks were made of polypropylene, while the experimental tanks were made of acrylic. A DO meter (OX11875) and DO sensors (OX11250) were bought from Loligo Systems (Viborg, Denmark). The pumps (MP-30R) were produced by Wenzhou Xinxishan Co., Ltd (Wenzhou, China). The solenoid valves (Q22MD-L8) were produced by Dalian Fangda Pneumatic Component Co., Ltd (Dalian, China). The water chillers, heaters (HSLS1000R Series, 660 kW), and custom-made purifiers were provided by Dalian Huixin Titanium Equipment Development Co., Ltd (Dalian, China).

### Experiment 1: Responses under moderate and severe hypoxia

For the first 20-day hypoxia tolerance experiment, four DO concentrations were set: 0.5, 1.0 and 2.0 mg L^-1^ for the treatment groups, and 6.0 mg L^-1^ for the control group, corresponding to the four environmental simulation systems of the device (salinity 30.0 ± 1.0; water temperature 22 ± 0.5°C; flow rate 250 L h^-1^). Each clam selected from the holding aquarium was marked with an individual number on the shell before being placed into an experimental tank. Every 96 clams were randomly assigned to each of the four groups, with 24 clams assigned to each of four replicate tanks. Three tanks (tanks A, B and C) were intended for observations of survival and behavior, as well as the analyses of metabolism and cellular damage performed at the end of the experiment; one tank (tank D) was intended for metabolism research on the 10^th^ day of the experiment. The experimental tanks were covered with 2-mm-thick black canvas to block out sunlight. All clams could bury themselves and adapt to the environment for 96 hours. During the first 72 hours, we replaced any clams that died or did not bury, while during the last 24 hours we only removed the dead or unburied ones without making replacements. To check if a clam is dead, we touched the siphons or foot three times with a glass rod. If the clam showed no response, it was judged to be dead. All handling was completed at least 8 hours before the experiment commenced. The animals were not fed during the acclimation period and throughout the whole experiment.

The DO concentration was maintained at 6.0 mg L^-1^ during the 96-hour adaptation period and thereafter reduced to the target (treatment) values within 48 hours. During the experiment, mortalities and the number of clams emerging from the sediment in tanks A, B and C were monitored every 8 hours, while DO was monitored daily using the Winkler method, and pH was measured at the beginning and end of the experiment. In addition, to maintain quality of the seawater, one-third was replaced every 3 days.

On the 10^th^ day of the experiment, 8 clams were chosen randomly from tank D for measurements of OCR and ammonia-N excretion rate (AER) as well as for determination of the O:N ratio, and another 8 clams were chosen for enzyme activities analysis.

At the end of the experiment, 19 clams were chosen randomly from tanks A, B and C. Of these, 8 clams were used for the measurement of OCR and AER as well as determination of the O:N ratio; 8 clams were used for enzyme activity analysis; and 3 clams were prepared for study under a transmission electron microscope (TEM). To ensure randomness of sampling and mitigate potential bias, a random-number software was used, and the clams were removed according to the number on their shell. To collect clams still buried in the sediment and to avoid excessive disturbance to them, a low-power pump was used to suck the sediment away, layer by layer. After the buried clams were exposed, they were carefully removed for the given analysis.

To measure the OCR, AER and O:N ratio, each chosen clam was immediately moved from the experimental tank to a 1-L sealed beaker of water. The beaker was warmed by a 22°C water bath and covered with 2-mm-thick black canvas. Seawater stored in the supply tank was used as the source water for the corresponding group of clams, except that the clams exposed to 0.5 mg L^-1^ DO were analyzed using the source water from the 1.0 mg L^-1^ DO supply tank, out of concern that a starting DO concentration of 0.5 mg L^-1^ might be too low to control for error in the OCR measurement. The source water was sterilized by ultraviolet lamp for 24 hours before use. The concentrations of DO and ammonia-N were measured at the beginning and 1 hour later. The calculation formulas were as follows:

$$OCR=\frac{{DO}_{0}-{DO}_{t}}{W\times T}$$

$$AER=\frac{N_{0}-N_{t}}{W\times T}$$

$$O:N=\frac{OCR}{AER}$$

where DO_0_ and N_0_, respectively, refer to the concentrations of DO and ammonia-N at the beginning of the experiment, and DO_t_ and N_t_ are the concentrations at the end; *W* is the dry weight of the clam, and *T* is the duration of the measurement; the O:N ratio reflects the composition of metabolic fuel [28]. An O:N ratio with a value of 7 indicates that energy will be provided by protein; the higher the value, the more the energy supply is derived from fats and carbohydrates instead of protein [29].

For measures of enzyme activity, the activities of lactate dehydrogenase (LDH), phosphofructokinase (PFK), and pyruvate kinase (PK) in adductor muscle were determined spectrophotometrically by monitoring the absorbance change at 340 nm caused by the oxidation of NADH. The adductor muscles were cut into roughly 3 × 3 × 3 mm sections and immediately stored in liquid nitrogen. Commercial assay kits (the total protein assay kit A045-4, lactate dehydrogenase assay kit A020-2, phosphofructokinase test kit A129 and pyruvate kinase assay kit A076-1; Nanjing Jiancheng Bioengineering Institute, Nanjing, China) were used according to the manufacturer’s instructions. The experimenter responsible for the measurements was blinded to the treatment origin of the clams as the centrifuge tubes were renumbered after sampling by another experimenter.

For observations with the TEM (JEM-1200EX; JEOL, Tokyo, Japan), foot muscles were sectioned into roughly 2 × 2 × 3 mm pieces and immediately fixed with 1 mL of 2.5% glutaraldehyde fixative before being stored in a refrigerator at 4°C. All samples were renumbered and sent to the Medical College of Qingdao University (Qingdao, China) for cell and organelle structure analysis within 2 hours; the experimenters were blinded from any information about the experiment design as well as the clam treatment groups.

Before Experiment 1, we had carried out another two pre-experiments on survival and behavioral response with the same experimental design as Experiment 1. One of the pre-experiments was terminated on the 15^th^ day for accidental electricity cut off.

### Experiment 2: Survival rates within the key DO concentration range

To further explore the survival rate against DO concentration, especially the key DO concentration range that corresponded to a drastic change in the survival rate, another set of 20-day tolerance experiments was carried out. The DO concentrations were set based on the survival results of Experiment 1, and 6.0 mg L^-1^ was adopted for all control groups. Each experiment involved 96 randomly chosen clams in each group, with 24 clams in each of four replicate tanks (Table 2). Other environmental conditions were kept the same as in Experiment 1 (salinity 30.0 ± 1.0; water temperature 22 ± 0.5°C; flow rate 250 L h^-1^). All tanks were covered with 2-mm-thick black canvas. Before the start of the experiments, all clams were allowed to adapt for 96 hours. As before, we replaced any dead or unburied clams found during the first 72 hours, but only removed these without planting new ones during the following 24 hours. The animals were not fed during the adaption periods and the whole experiments. Mortalities were checked daily and DO was monitored by the Winkler method. One-third of the seawater was replaced every three days, as in Experiment 1.

**Table 2:** Dissolved oxygen (DO) concentrations used in Experiment 2.

Experiment number	Treatment groups (3 tanks) DO (mg L^-1^)	Control group (1 tank) DO (mg L^-1^)	Replicates	Date
2.1	0.40, 0.60, 0.80	6.0	4	March 10 to April 1, 2018
2.2	0.45, 0.65, 0.85	6.0	4	May 5 to 27, 2018
2.3	0.50, 0.70, 0.90	6.0	4	June 15 to July 7, 2018
2.4	0.55, 0.75, 0.95	6.0	4	April 7 to 29, 2018

### Statistical analyses

OCR, AER, O: N and all measures of enzyme activity were tested for statistical significance. To ensure preciseness of the conclusions, all data were first tested for normality and homogeneity of variances. If the results passed all two tests, analysis of variance (ANOVA) would be adopted, and a Tukey test was used for multiple comparisons. If the results failed to pass normality tests or homogeneity of variances tests, the Kruskal–Wallis H-test was adopted, and multiple comparisons were conducted with the Mann–Whitney U-test. The numbers of surviving and buried clams were analyzed using the latter method, as nonparametric statistical methods are more reliable (although less sensitive) than ANOVA when values for replicates are not large enough (Supporting information). No difference being introduced by hypoxia was given as the null hypothesis and a *p*-value of < 0.05 defined statistical significance of the results.

To better control the potential deviation caused by other factors, we made a comparison across Pre-experiment 1, Pre-experiment 2 and Experiment 1. The calibrated values (CV) of survival rates and burial rates for each group in each experiment was defined as follows:

$${CV}_{survival\;rate}=\frac{n_{2}}{n_{1}}\times \frac{N_{1}}{N_{2}}$$

$${CV}_{burial\;rate}=\frac{n_{3}}{n_{2}}\times \frac{N_{2}}{N_{3}}$$

where n_1_ and n_2_, respectively, refer to the initial number of surviving clams in one specific group and the number of surviving clams on the 15^th^ day in the same group; N_1_ and N_2_, respectively, refer to the initial number of surviving clams in the corresponding control group and the number of surviving clams on the 15^th^ day of the same control group; n_3_ and N_3_, respectively, refer to the number of buried clams in the specific group and the corresponding control group mentioned above. The CV were then compared across different DO concentrations, using Kruskal–Wallis H-test.

The 20-day LC_50_ for 0.5 mg L^-1^ DO and the LT_50_ were determined using probit analysis [30]. All statistical analyses were carried out with SPSS 16.0 (IBM, Armonk, New York State, United States of America).

## Results

### Survival and behavioral response

**Fig 2:**
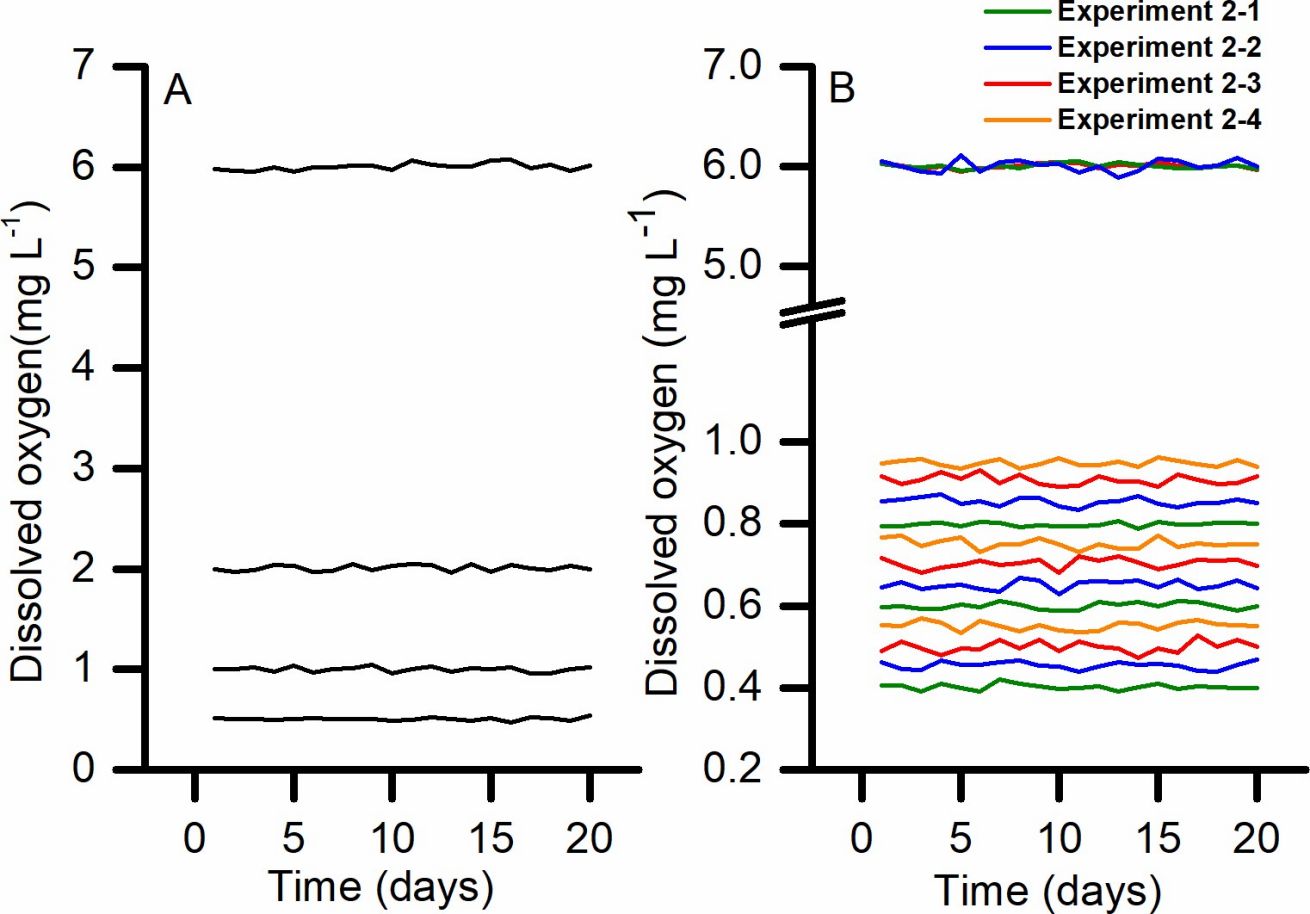
Dissolved oxygen (DO) treatments used in Experiments 1 and 2; the control group in each experiment was maintained at 6.0 mg L^-1^ DO. (A) Experiment 1 consisted of three treatment groups, set at 0.5, 1.0 and 2.0 mg L^-1^ DO, and a control group. (B) Experiment 2 comprised four independent experiments with different sets of treatments: Experiment 2.1 –treatments 0.40, 0.60 and 0.80 mg L^-1^ DO, and a control group; Experiment 2.2 –treatments 0.45, 0.65 and 0.85 mg L^-1^ DO, and a control group; Experiment 2.3 –treatments 0.50, 0.70 and 0.90 mg L^-1^, and a control group; Experiment 2.4 –treatments 0.55, 0.75 and 0.95 mg L^-1^, and a control group.

The experimental DO concentrations were accurately and stably controlled by the automated hypoxia simulation device during both experiments, which ensured reliability of the results (Fig 2). The measures of DO concentration levels in all the experiments were attached to the Supporting information.

**Fig 3:**
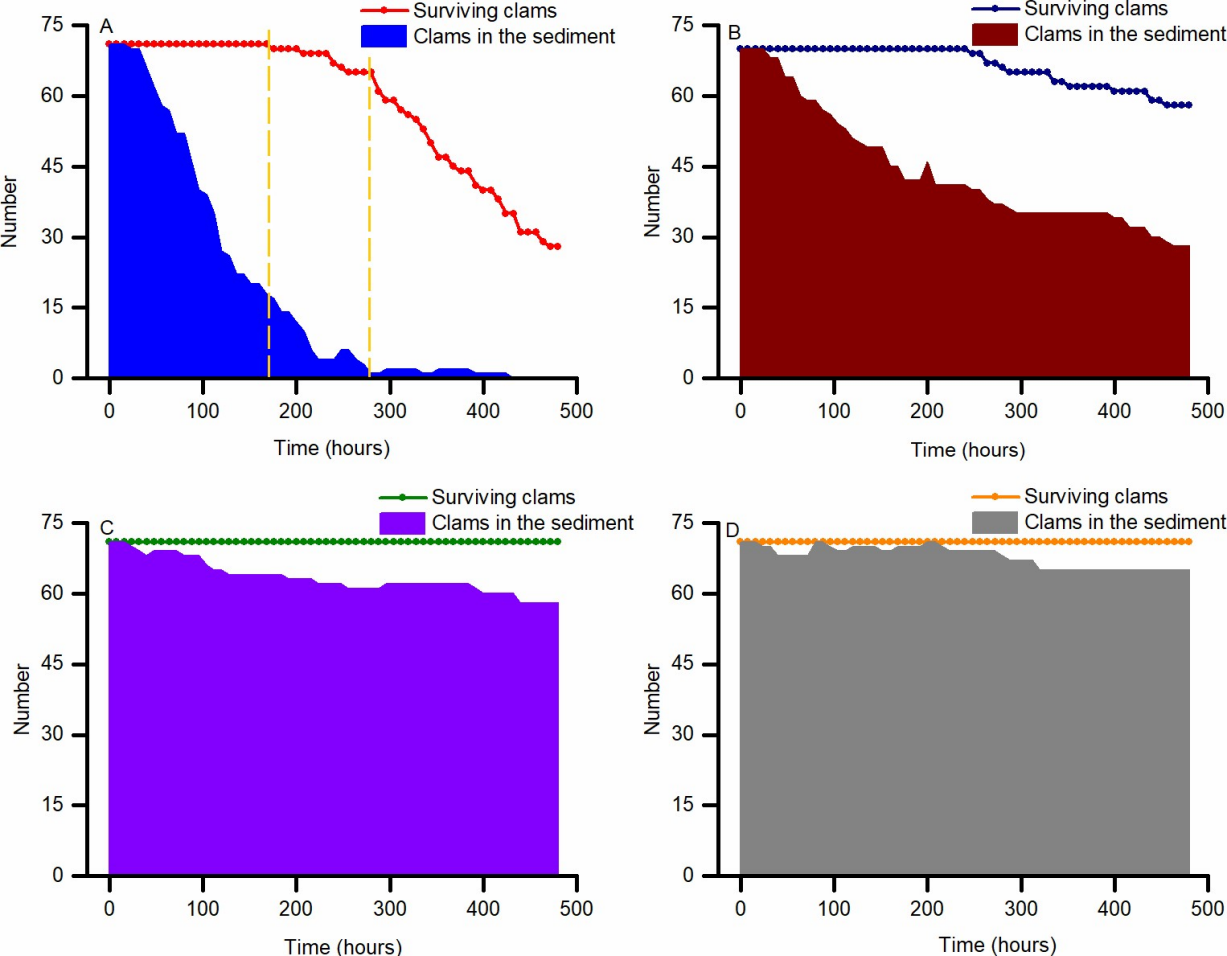
Changes in the number of surviving and buried *Ruditapes philippinarum* in Experiment 1. (A) Treatment group exposed to 0.5 mg L^-1^ DO. The dashed lines denote the estimated critical-time thresholds dividing the curve into different phases; the first dashed line marks the beginning of the mortalities, and the second cuts the rest of the curve into two parts that could each be linearly fitted with best fitness (the largest sum of R values in Excel). (B) Treatment group exposed to 1.0 mg L^-1^ DO. (C) Treatment group exposed to 2.0 mg L^-1^ DO. (D) Control group maintained at 6.0 mg L^-1^ DO. *N* = 72 per treatment.

**Fig 4:**
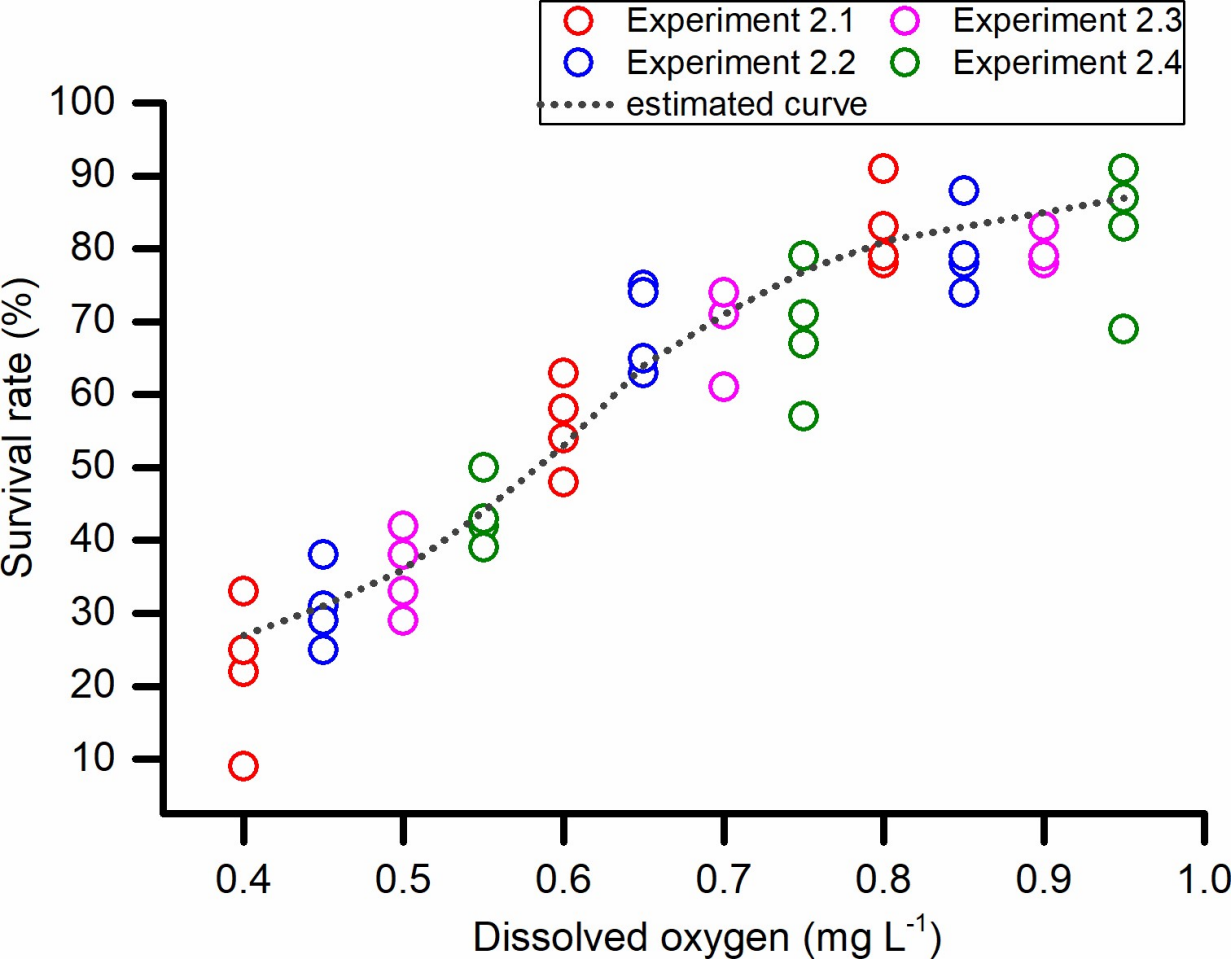
Survival rates of the *Ruditapes philippinarum* in Experiment 2. Each circle represents one replicate in one specific experiment. The 20-day LC_50_ for DO was estimated by probit analysis, based on the combined results of Experiments 2.1–2.4. *N* = 96 per treatment.

Hypoxia poses a great threat to the survival of Manila clam. However, mortalities only occurred under the low DO concentrations of 0.5 and 1.0 mg L^-1^, while a moderately hypoxic environment of 2.0 mg L^-1^ did not cause any clams to die (Fig 3). During the first 7 days of exposure, at all DO concentrations, the clams displayed strong tolerance to hypoxia as no mortalities were recorded. During the next 3 to 5 days, a few of the clams exposed to 0.5 mg L^-1^ DO began to die at a slow rate, followed by deaths among those exposed to 1.0 mg L^-1^ DO. The survival rate decreased sharply among clams exposed to the most severe hypoxia (0.5 mg L^-1^ DO), resulting in much lower survival among these than the clams exposed to 1.0 mg L^-1^ DO (*p* = 0.05). The LT_50_ at 0.5 mg L^-1^ DO was calculated as 422 hours, and the 20-day LC_50_ was estimated to be 0.57 mg L^-1^ DO when the experiments using more DO concentrations were conducted as part of Experiment 2. When the DO concentration was decreased from 1.0 to 0.4 mg L^-1^, survival dropped from nearly 90% to approximately 20%; survival began to drop more suddenly around 0.8 mg L^-1^ DO and dropped most dramatically at about 0.6 mg L^-1^ DO (Fig 4).

As shown in Table 3, the variances between the results of Experiment 1 and the two Pre-experiments were small after calibration by control group, and the effects of hypoxia on survival and behavior were still significant, suggesting that there was little chance that Experiment 1 was affected by a potential factor.

**Table 3:** Calibrated values (CV) for survival rates and burial rates of the *Ruditapes philippinarum* (* indicates *p* < 0.05).

Dissolved oxygen (mg L^-1^)	Experiment number	CV for survival rates on the 15^th^ day		CV for burial rates on the 15^th^ day	
0.5	Pre-experiment 1	0.63	A significant difference was detected (Kruskal–Wallis H-test, H = 10.173, *p* = 0.017).	0.02	A significant difference was detected (Kruskal–Wallis H-test, H = 10.532, *p* = 0.015).
	Pre-experiment 2	0.65		0.09	
	Experiment 1	0.66		0.05	
	Mean value	0.65*		0.05*	
1.0	Pre-experiment 1	0.85		0.55	
	Pre-experiment 2	0.93		0.64	
	Experiment 1	0.89		0.62	
	Mean value	0.89*		0.60*	
2.0	Pre-experiment 1	1.03		0.88	
	Pre-experiment 2	1.00		0.94	
	Experiment 1	1.00		0.95	
	Mean value	1.01		0.92*	
6.0	Pre-experiment 1	1.00		1.00	
	Pre-experiment 2	1.00		1.00	
	Experiment 1	1.00		1.00	
	Mean value	1.00		1.00	

As a major behavioral response towards hypoxia, the Manila clams emerged from the sediment in their tanks to obtain more oxygen; at the lower DO concentrations, the less the clams buried (Fig 3). However, the clams that were successively exposed to all DO concentrations seemed to reach a relatively stable condition around the 9^th^ day, even after dramatic changes in burial rates. All clams exposed to 0.5 mg L^-1^ DO emerged at the surface of the sediment, which suggests this level of hypoxia is an extremely challenging environment for the species.

### Metabolic response

**Fig 5:**
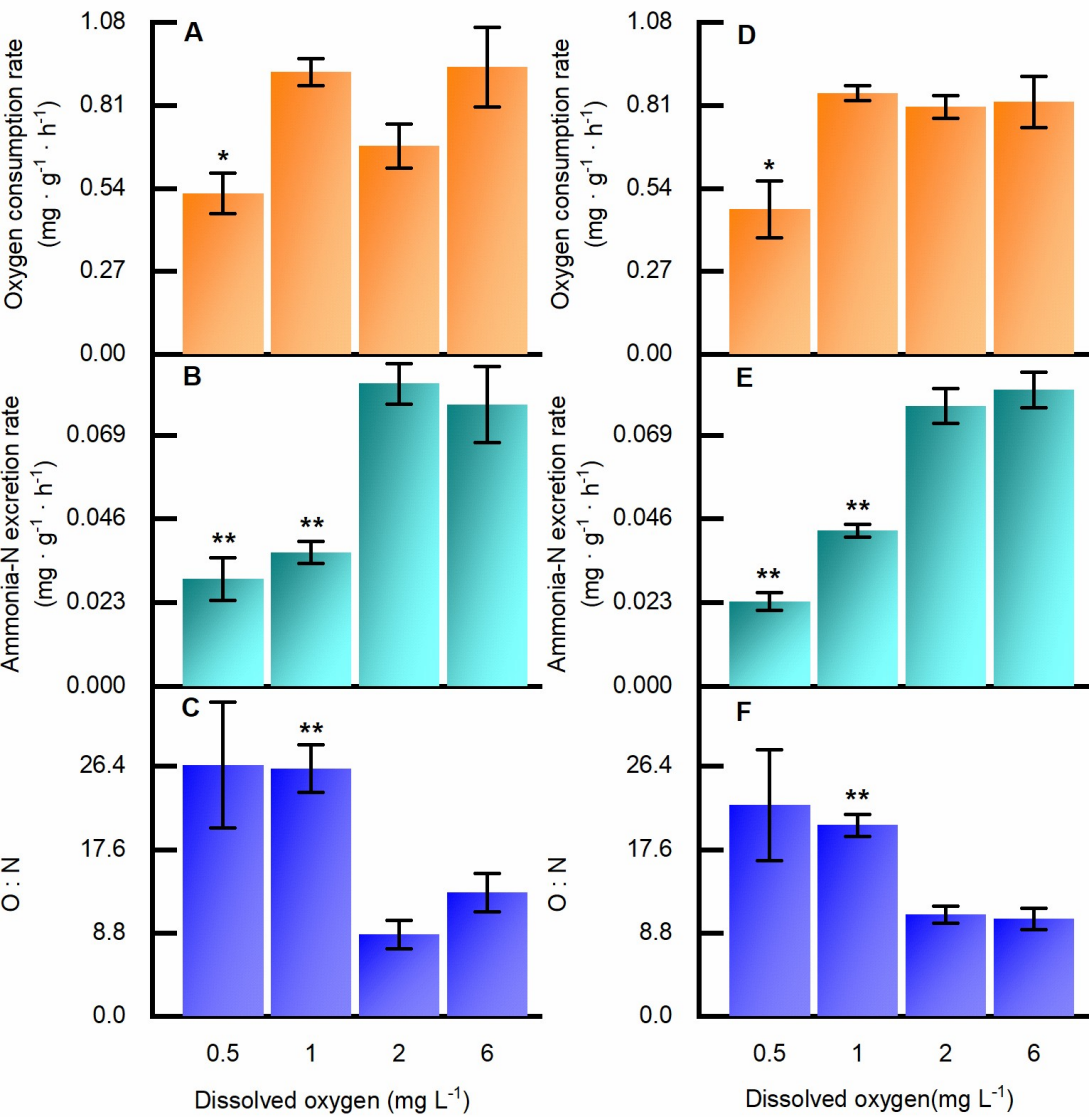
Metabolic response to hypoxia by *Ruditapes philippinarum* on the 10^th^ day and at the end of the experiment, under different DO concentrations. (A) Oxygen consumption rate on the tenth day; (B) ammonia-N excretion rate on the tenth day; (C) O:N ratio on the tenth day. (D) oxygen consumption rate at the end of experiment; (E) ammonia-N excretion rate at the end of experiment; (F) O:N ratio at the end of experiment. Error bars indicate mean ± SE (** indicates *p* < 0.01, * indicates *p* < 0.05).

OCR, AER, and the O:N ratio were significantly influenced by hypoxia. However, these values indicated a response only to certain DO concentrations, especially the lower concentrations (Fig 5). By the end of the experiment, no difference in OCR was detected between the clams exposed to 1.0, 2.0 and 6.0 mg L^-1^ DO (*p* = 0.878), whereas the clams exposed to 0.5 mg L^-1^ DO exhibited distinct respiratory depression (*p* = 0.022). For AER, a significant suppression effect under hypoxia could be observed when the DO concentration dropped to 1.0 mg L^-1^ and below. The clams exposed to 2.0 and 6.0 mg L^-1^ DO did not differ significantly in AER (*p* = 0.85). The O:N ratio rose sharply when the DO dropped from 2.0 to 1.0 mg L^-1^ and below. There was no significant difference between the results for clams removed on the 10^th^ day and at the end of the experiment, whether for values of OCR, AER or O:N (*p* > 0.05).

**Fig 6:**
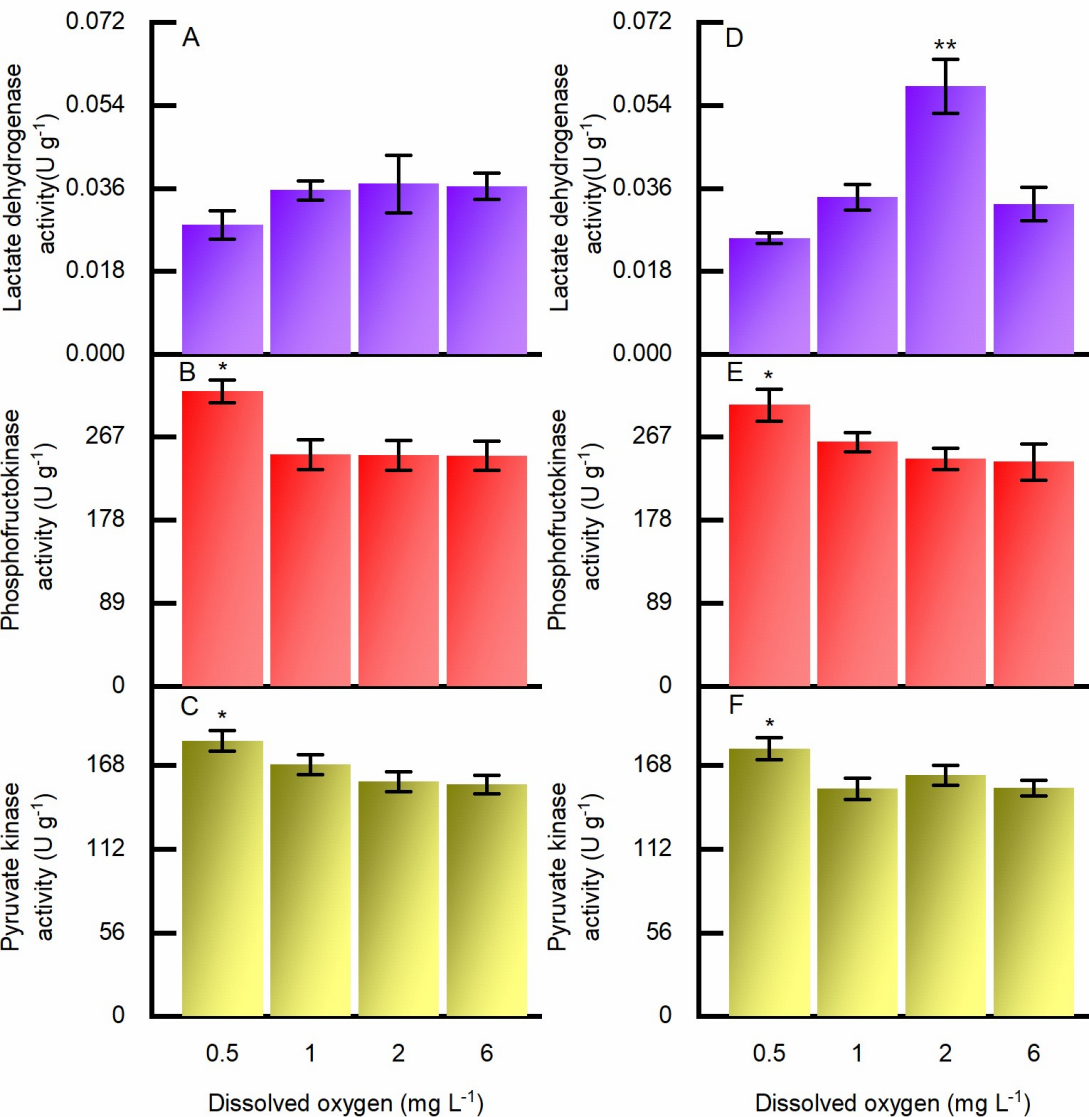
Biochemical responses to hypoxia measured in adductor muscle of *Ruditapes philippinarum* on the 10^th^ day and at the end of the experiment, under different DO concentrations. (A) Lactate dehydrogenase activity on the tenth day; (B) phosphofructokinase activity on the tenth day; (C) pyruvate kinase activity on the tenth day. (D) lactate dehydrogenase activity at the end of experiment; (E) phosphofructokinase activity at the end of experiment; (F) pyruvate kinase activity at the end of experiment. Error bars indicate mean ± SE (** indicates *p* < 0.01, * indicates *p* < 0.05).

The effects of hypoxia on LDH, PFK, and PK activities in adductor muscle were significant. Compared with the response mode of PK and PFK, whose activities peaked under the most severe hypoxia (0.5 mg L^-1^ DO); LDH reached its highest activity at the DO concentration of 2 mg L^-1^ (Fig 6). The depression of LDH activity when the DO dropped from 2.0 to 1.0 mg L^-1^ may suggest diversified pathways of pyruvate. The results on the 10^th^ day did not differ from those at the end of the experiment (*p* > 0.05), except that LDH activity did not peak at the DO concentration of 2.0 mg L^-1^.

### Cellular damage

**Fig 7:**
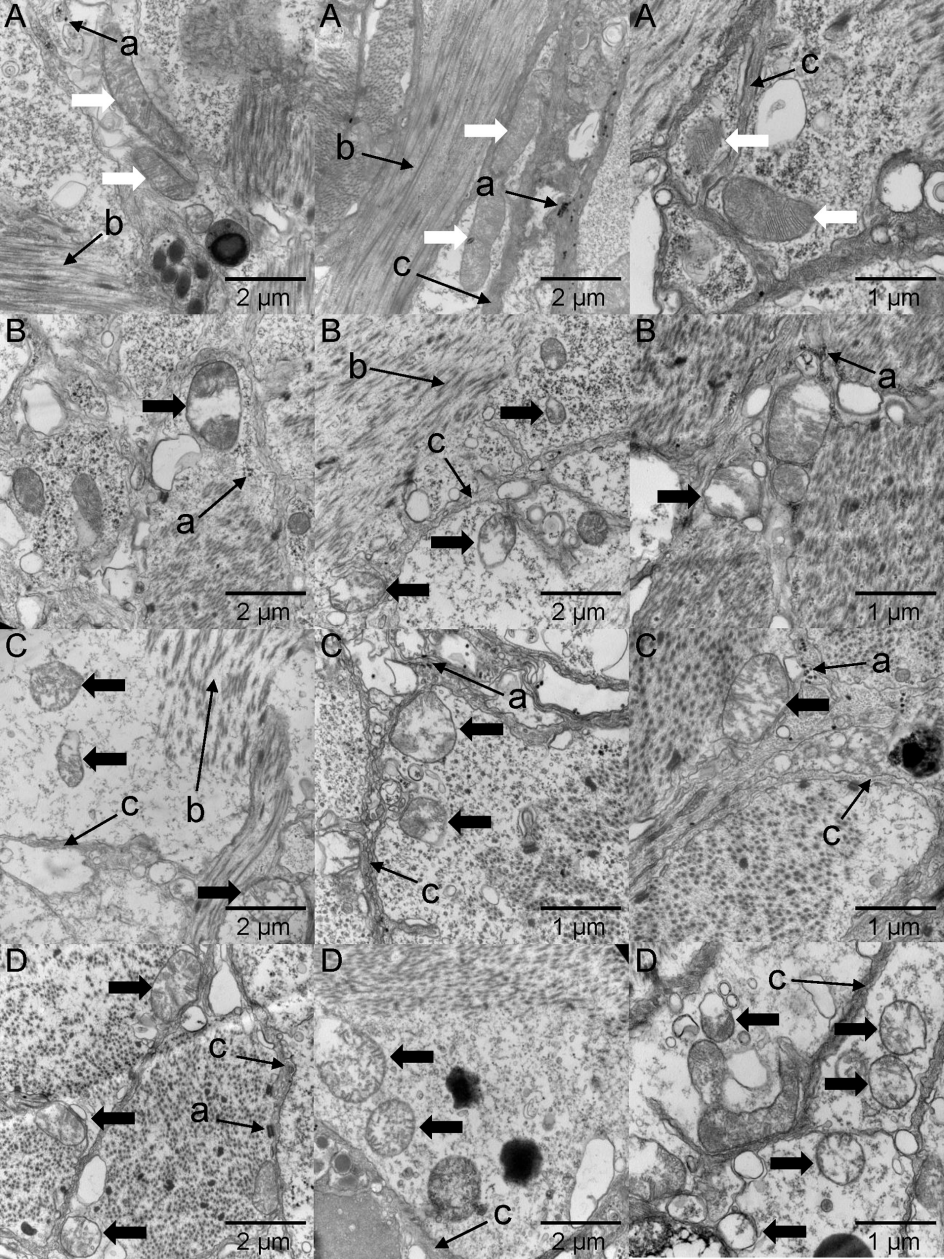
Transmission electron microscope images of the foot muscle of *Ruditapes philippinarum*. (A), (B), (C) and (D) represent the DO groups of 6.0, 2.0, 1.0 and 0.5 mg L^-1^, respectively. White arrows point to normal mitochondria while black arrows point to mitochondria with collapsed cristae (judged by sparse cristae) and vacuolization (judged by shallower staining). Cell vacuolization was apparent according to the shallower cell-staining. Some other cellular structures for point of reference are listed as follows: ‘a’ refer to glycogen granules; ‘b’ refer to myofilaments; ‘c’ refer to cell membrane.

Under hypoxic stress, damage was observed at the cellular level in the Manila clams (Fig 7). Mitochondria, the main site of aerobic respiration for cells, were damaged to various degrees, with collapsed cristae, shriveled membranes and induced cell inclusion. Though the cell remained intact with no membrane rupture, vacuolization was apparent from the lighter staining of chromatin. Other organelles seemed to present a normal appearance except that the myofilaments tended to dissolve.

## Discussion

### Survival and behavioral response

Tolerance to hypoxia can vary widely among different aquatic taxa [31]. Fishes are considered the most sensitive, with a high minimum oxygen requirement, followed by crustaceans and echinoderms, while shellfish are the most tolerant [20]. The LC_50_ for DO and the LT_50_ at 0.5 mg L^-1^ DO indicate a strong tolerance to hypoxia by Manila clam, even compared with other shellfish [19]. Hence, these bivalves are more likely to survive a moderately hypoxic episode than other species, and thus the species may assume a dominant position in the marine and brackish macrobenthic community.

The results presented here may prove useful for farming of this species as no mortalities occurred at 2.0 mg L^-1^ DO, a concentration widely reported to occur in mariculture areas [15]. For example, where it may not be advisable to raise scallops in coastal regions threatened by oxygen deficiency, the culture of Manila clams may still be feasible and comparatively profitable. In terms of severe hypoxia, the fate of Manila clams may depend on the duration of the event. Unlike many other clam species, such as the widespread *Macoma balthica* which begin to die immediately in hypoxic conditions [32], there might be two time thresholds for Manila clam, as shown in Fig 3. The observation of a period of tolerance followed by a slow rate of mortalities and then sudden mortalities indicates that a hypoxic episode lasting under a week or one lasting more than one week can produce totally different outcomes for this species.

Furthermore, when the survival rate was plotted against DO concentrations below 1.0 mg L^-1^ in Experiment 2, we found that the curve was S-shaped rather than a straight line, implying that subtle differences in DO during a severely hypoxic event could result in a different fate for Manila clams. The ability of Manila clam to endure hypoxia may reach a limit when DO drops below 0.8 mg L^-1^, and such low DO concentrations may result in rapid collapse of the population.

The behavioral response of the clams was quite immediate as the treated clams began to emerge from the sediment within 48 hours. Under severe hypoxia (0.5 mg L^-1^), the number of animals remaining buried reduced sharply and all of them emerged afterwards. These results support the hypothesis that organisms facing hypoxia will first find ways to ensure their oxygen supply before turning to anaerobic respiration [33]. However, tests with clams in other kinds of sediments might yield different results because of differences in breathability and variations in the organic contents which might influence the abundance of aerobic microorganisms. The ecological consequences of shellfish emergence in shallow coastal areas have been widely discussed, and most researchers believe this increases the species’ risk of becoming prey [34,35]. Further research should be conducted to consider other factors like the tolerance to hypoxia by predators, and hypoxia strength and recovery times.

### Metabolic response

Metabolic strategies under conditions of insufficient oxygen are vital for keeping an organism alive. Stickle et al. (1989) found that the metabolic rates of several species of crustaceans and mollusks were depressed under hypoxia, as reflected in their rates of heat dissipation [36]. For Manila clam, metabolic depression under hypoxia was obvious, as revealed by the lower values of OCR and AER. An animal’s natural response is to reduce energy expenditure as anaerobic fermentation runs at low efficiency [22]. Before we carried out these experiments, we had expected that the depression of OCR would positively correlate with decreasing DO. Surprisingly, depressed OCR was only observed under the most severely hypoxic condition (0.5 mg L^-1^ DO), while other treatment groups exhibited no significant differences; this result may provide us clues about the species’ strong tolerance. Compared with less-tolerant bivalves whose OCR decrease under moderate hypoxia [37], the Manila clam exhibited a progressive oxygen regulatory capacity, reflected in the relatively constant values of OCR, even at 1.0 mg L^-1^ DO. Combined with its strong tolerance to a wide range of water temperatures as well as salinities [38], the Manila clam can endure some of the most challenging shallow coastal environments. Slightly different from the values for OCR, AER was depressed in both the 0.5 and 1.0 mg L^-1^ DO treatment groups, providing evidence that, in Manila clam, protein catabolism is sensitive to reduced oxygen levels.

Based on the results of OCR and AER, the O:N ratio was about 10 under the DO concentrations of 2.0 and 6.0 mg L^-1^, but the ratio rose to around 20 when DO dropped to 1.0 mg L^-1^ and below. Thus, it is likely that a major shift of energy supply happened as carbohydrates and fats became the preferred elements. This shift suggests that energy-releasing efficiency could be important as fats and carbohydrates are the most efficient nutrients for energy storage. Considering that feeding would become depressed in a hypoxic environment [33], the clams with more fat reserves may have a better chance of surviving.

To survive hypoxic challenges, a depressed metabolism itself is not sufficient. In the early stage of hypoxia, the breakdown of phosphagens and the transamination of aspartate coupling with glycolysis can serve as major pathways for energy production in invertebrates, leaving succinate and alanine as end products [39–41]. But to survive long-term oxygen deficiency, glycogen is one of the most crucial energy sources [42]. PFK, the key enzyme in glycolysis regulation (which was expected to be depressed, according to some previous studies with mollusks), showed surprisingly increased activity as the DO concentration dropped to 0.5 mg L^-1^, suggesting existence of the Pasteur effect (i.e., an increased glycolytic rate during anoxia despite depressed metabolism) in Manila clam. The possible reason why PFK activity rose when metabolism should be depressed may be that the metabolic intensity of the adductor muscle is higher than the average metabolic intensity of Manila clam. Lushchak et al. (1998) found that under hypoxia, PFK activity in the high-energy-consuming brain of the black scorpionfish *Scorpaena porcus* increased by 56% though it decreased significantly in the liver [43]. Thus, it could be inferred that the adductor muscle of the Manila clam, responsible for opening and closing the shell, may consume more energy, exceeding the output of a depressed metabolism and resulting in the upregulation of key enzyme activities in carbohydrate metabolism. Furthermore, the duration of hypoxia may play a role. Nile tilapia, *Oreochromis niloticus*, an extremely hypoxia-tolerant fish, had decreased PFK activity in its white muscle and liver after one day of exposure to hypoxia, yet the activity of PFK increased when the duration was prolonged to 30 days [44]. PK, another rate-limiting enzyme responsible for the reaction from phosphoenolpyruvate to pyruvate, displayed a similar trend in activity change. It has been widely revealed that under hypoxic conditions phosphoenolpyruvate can be catalyzed to oxaloacetate instead of pyruvate with the inhibition of PK and the activation of phosphoenolpyruvate carboxykinase [45,46]. In the present study, PK activity in Manila clam showed no sign of inhibition but increased under the lowest DO concentration, suggesting that the pyruvate pathway may be preferred to the succinate pathway in situations of long-term exposure to severe hypoxia.

To keep anaerobic glycolysis running, NADH generated from oxidation reactions must be reoxidized, and LDH (the classic pyruvate reductae) is prevalent among different crustaceans, echinoderms and some mollusks [47,48]. The LDH-activity response was quite different from the other enzymes assessed, as it reached a maximum value under the DO concentration of 2.0 mg L^-1^ at the end of the experiment and declined with either an increase or decrease of DO. One possible explanation is that since pyruvate is catalyzed by at least five more reductases, which are usually called opine dehydrogenases, resulting in other end products, such as octopine, strombine, alanopine and tauropine [49,50], LDH inactivity under severe hypoxia may indicate a diversified reaction pathway. It is likely that alternative end products to lactate were of great significance to maintain cellular homeostasis, since the accumulation of acid is largely avoided.

### Cellular damage

Tissue damage under hypoxic conditions has been previously demonstrated for Manila clam [51], thus this study focused on damage at the cellular level. Previous studies reported that the mitochondria of marine mollusks have strong resilience against environmental hypoxia, as reflected mainly in substrate oxidation, phosphorylation and proton leak [52–54]. However, we could not find any study of marine organisms that focused on structural responses of the mitochondria to hypoxia. In the present study, we were able to detect cellular changes at an ultrastructure level by examination with TEM. Compared with the clams in the control groups, long-term hypoxia caused the collapse of mitochondrial cristae. Since the cristae are a crucial place for respiratory enzymes to attach as well as a location where reactions proceed, this damage may be ultimately responsible for death of the clam. Vacuolization in both the cells and mitochondria may indicate cellular dysfunction, considering that vacuolization in animal cells is related to pathology. Furthermore, the reoxygenation process may cause other injuries [55], leaving the clams vulnerable to other environmental stresses or fluctuations. Thus, an important direction for future research might be to study the post-effects of hypoxia on Manila clam to better understand the complete ecological consequences.

### Finding a reliable test environment to provide high DO accuracy and stability

In experiments focusing on the effects of hypoxia on marine organisms, especially tolerance assessments, the approach to creating the hypoxic environment will affect the credibility of the results [19,56]. Simply bubbling nitrogen into an airtight chamber of water has been widely adopted for its simplicity and cheapness. However, the nitrogen flow itself is somewhat of a shock to the animals, and DO fluctuations caused by the time delay may eventually affect the results [57]. Over the last two decades, *in situ* experiments have begun to prevail. With experimental chambers placed on the seabed instead of setting up in the laboratory, less error is generally introduced by environmental factors. But most *in situ* devices designed to date only have the capacity to simulate the transition from normoxia (normal levels of oxygen) to anoxia by sealing the chambers, making it impossible to maintain precise DO concentrations [58,59].

As our results show, the newly designed automated device reliably controlled the set DO concentrations precisely and accurately, owing to the device’s huge capacity, the water circulating feature via the supply tank, the luminescence-quenching DO sensors that enabled continuous measurements, and the hysteresis principle we established to eliminate the gap between the DO sensor’s response time and the mixing of the water. In addition, water-quality control was given special attention in design of the device, as the purifier together with the water-exchange system effectively reduced the concentrations of organic and inorganic impurities.

## Conclusions

To investigate long-term tolerance to hypoxia by the commercially important Manila clam, a series of laboratory experiments were carried out. This bivalve’s responses in terms of mortality, behavior and metabolism under a wide range of DO concentrations were determined in the context of the 20-day LC_50_, respiratory enzyme activities, and cellular damage. A novel hypoxia simulation device was used to ensure the accuracy and stability of the experimental environment. The implications of the results are as follows: (1) Moderate or short-term hypoxia does not threaten the survival of Manila clam; however, their behavioral response of emerging from the sediment to get more oxygen could increase their chance of being preyed upon by hypoxia-tolerant predators. (2) As a consequence of a strongly depressed metabolism under severe hypoxia, energy would be consumed first by basic life-sustaining activities, thus the animal’s growth and reproduction may be inhibited. (3) Manila clam may have a different strategy for glycolysis regulation, as the activities of PFK and PK in adductor muscle showed an unexpected rise after exposure to severe hypoxia, a finding that differs from determinations for many other hypoxia-tolerant species. Finally, (4) Manila clams that survive a hypoxic environment may subsequently suffer from further oxygen fluctuations, pathogens, or other environmental stresses due to collapse of the mitochondrial cristae and vacuolization in both the cells and the mitochondria.

## Supporting information

S1 Table Detailed statistical analyses information. (DOCX)

S2 Table Measures of dissolved oxygen concentration levels. (DOCX)

S1 Data Experimental data in this study. (ZIP)

## References

[1] Diaz RJ, Rosenberg R. Spreading dead zones and consequences for marine ecosystems. Science. 2008;321(5891):926–9. 10.1126/science.1156401 18703733

[2] Levin LA, Breitburg DL. Linking coasts and seas to address ocean deoxygenation. Nature Climate Change. 2015;5(5):401 10.1038/nclimate2595

[3] Sugden AM. Threats of coastal hypoxia. Science. 2017;356(6333):38 10.1126/science.356.6333.38-a 28385997

[4] Vaquer-Sunyer R, Duarte CM. Thresholds of hypoxia for marine biodiversity. Proceedings of the National Academy of Sciences. 2008;105(40):15452–7. 10.1073/pnas.0803833105 PMC2556360 18824689

[5] Condon RH, Duarte CM, Pitt KA, Robinson KL, Lucas CH, Sutherland KR, et al Recurrent jellyfish blooms are a consequence of global oscillations. Proceedings of the National Academy of Sciences. 2013;110(3):1000–5. 10.1073/pnas.1210920110 PMC3549082 23277544

[6] do Rosário Gomes H, Goes JI, Matondkar S, Buskey EJ, Basu S, Parab S, et al Massive outbreaks of *Noctiluca scintillans* blooms in the Arabian Sea due to spread of hypoxia. Nature Communications. 2014;5:4862 10.1038/ncomms5862 25203785

[7] Diaz RJ, Rosenberg R. Marine benthic hypoxia: a review of its ecological effects and the behavioural responses of benthic macrofauna. Oceanography and Marine Biology: an Annual Review. 1995;33:245–03.

[8] Zhang J, Gilbert D, Gooday A, Levin L, Naqvi S, Middelburg J, et al Natural and human-induced hypoxia and consequences for coastal areas: synthesis and future development. Biogeosciences. 2010;7:1443–67. 10.5194/bg-7-1443-2010

[9] Munari M, Matozzo V, Marin MG. Combined effects of temperature and salinity on functional responses of haemocytes and survival in air of the clam *Ruditapes philippinarum*. Fish & Shellfish Immunology. 2011;30(4–5):1024–30. 10.1016/j.fsi.2011.01.025 21315156

[10] Welsh DT, Nizzoli D, Fano EA, Viaroli P. Direct contribution of clams (Ruditapes philippinarum) to benthic fluxes, nitrification, denitrification and nitrous oxide emission in a farmed sediment. Estuarine, Coastal and Shelf Science. 2015;154:84–93. 10.1016/j.ecss.2014.12.021

[11] Zhang JH, Fang JG, Tang QS. The contribution of shellfish and seaweed mariculture in China to the carbon cycle of coastal ecosystem. Advance in Earth Sciences. 2005;3:359–65.

[12] Savini D, Occhipinti–Ambrogi A, Marchini A, Tricarico E, Gherardi F, Olenin S, et al The top 27 animal alien species introduced into Europe for aquaculture and related activities. Journal of Applied Ichthyology. 2010;26:1–7. 10.1111/j.1439-0426.2010.01503.x

[13] Zhang G. Clam Aquaculture Study: Science Press; 2010.

[14] Gu XL, Xu ZL. A review on the effects of hypoxia on aquatic animals in estuaries. Marine Fisheries. 2009;4:013.

[15] Liu J, Zang J, Zhao C, Yu Z, Xu B, Li J, et al Phosphorus speciation, transformation, and preservation in the coastal area of Rushan Bay. Science of the Total Environment. 2016;565:258–70. 10.1016/j.scitotenv.2016.04.177 27177132

[16] Meng CX, Deng CM, Yao P, Zhang XQ, Mi TZ, Chen HT, et al Dissolved oxygen in the Xiaoqinghe Estuary and adjacent waters. Marine Environmental Science. 2005;3:006.

[17] Kozuki Y, Yamanaka R, Matsushige M, Saitoh A, Otani S, Ishida T. The after-effects of hypoxia exposure on the clam *Ruditapes philippinarum* in Omaehama beach, Japan. Estuarine, Coastal and Shelf Science. 2013;116:50–6. 10.1016/j.ecss.2012.08.026

[18] Uzaki N, Kai M, Aoyama H, Suzuki T. Changes in mortality rate and glycogen content of the Manila clam *Ruditapes philippinarum* during the development of oxygen-deficient waters. Fisheries Science. 2003;69(5):936–43. 10.1046/j.1444-2906.2003.00710.x

[19] Long WC, Brylawski BJ, Seitz RD. Behavioral effects of low dissolved oxygen on the bivalve *Macoma balthica*. Journal of Experimental Marine Biology and Ecology. 2008;359(1):34–9. 10.1016/j.jembe.2008.02.013

[20] Miller D, Poucher S, Coiro L. Determination of lethal dissolved oxygen levels for selected marine and estuarine fishes, crustaceans, and a bivalve. Marine Biology. 2002;140(2):287–96. 10.1007/s002270100702

[21] Larade K, Storey KB. A profile of the metabolic responses to anoxia in marine. Sensing, Signaling and Cell Adaptation. 2002:27–46.

[22] Thomas Y, Flye-Sainte-Marie J, Chabot D, Aguirre-Velarde A, Marques GM, Pecquerie L. Effects of hypoxia on metabolic functions in marine organisms: Observed patterns and modelling assumptions within the context of Dynamic Energy Budget (DEB) theory. Journal of Sea Research. 2018;143:231–42. 10.1016/j.seares.2018.05.001

[23] Wang QN. The effects of hypoxia on the marine organisms in large-scale algal bloom area. M.Sc. Thesis, University of Chinese Academy of Sciences. 2012.

[24] Chen J, Mai K, Ma H, Wang X, Deng D, Liu X, et al Effects of dissolved oxygen on survival and immune responses of scallop (Chlamys farreri Jones et Preston). Fish & Shellfish Immunology. 2007;22(3):272–81. 10.1016/j.fsi.2006.06.003 16901718

[25] Cooper RU, Clough LM, Farwell MA, West TL. Hypoxia-induced metabolic and antioxidant enzymatic activities in the estuarine fish *Leiostomus xanthurus*. Journal of Experimental Marine Biology and Ecology. 2002;279(1–2):1–20. 10.1016/S0022-0981(02)00329-5

[26] Parrilla-Taylor DP, Zenteno-Savín T. Antioxidant enzyme activities in Pacific white shrimp (Litopenaeus vannamei) in response to environmental hypoxia and reoxygenation. Aquaculture. 2011;318(3–4):379–83. 10.1016/j.aquaculture.2011.05.015

[27] Tengberg A, Hovdenes J, Andersson HJ, Brocandel O, Diaz R, Hebert D, et al Evaluation of a lifetime‐based optode to measure oxygen in aquatic systems. Limnology and Oceanography: Methods. 2006;4(2):7–17. 10.4319/lom.2006.4.7

[28] Brown AC. Effect of natural and laboratory diet on O: N ratio in juvenile lobsters (Homarus americanus). Comparative Biochemistry and Physiology Part A: Molecular & Integrative Physiology. 2006;144(1):93–7. 10.1016/j.cbpa.2006.02.008 16549378

[29] Mayzaud P. Respiration and nitrogen excretion of zooplankton. II. Studies of the metabolic characteristics of starved animals. Marine Biology. 1973;21(1):19–28. 10.1007/BF00351188

[30] Finney D. Probit Analysis: Cambridge University Press; 2009.

[31] Rosenberg R, Hellman B, Johansson B. Hypoxic tolerance of marine benthic fauna. Marine Ecology Progress Series. 1991;79(1):127–31.

[32] Borsuk ME, Powers SP, Peterson CH. A survival model of the effects of bottom-water hypoxia on the population density of an estuarine clam (Macoma balthica). Canadian Journal of Fisheries and Aquatic Sciences. 2002;59(8):1266–74. 10.1139/f02-093

[33] Wu RS. Hypoxia: from molecular responses to ecosystem responses. Marine Pollution Bulletin. 2002;45(1–12):35–45. 10.1016/S0025-326X(02)00061-9 12398365

[34] Long WC, Seitz RD, Brylawski BJ, Lipcius RN. Individual, population, and ecosystem effects of hypoxia on a dominant benthic bivalve in Chesapeake Bay. Ecological Monographs. 2014;84(2):303–27.

[35] Seitz RD, Marshall L Jr, Hines A, Clark K. Effects of hypoxia on predator-prey dynamics of the blue crab *Callinectes sapidus* and the Baltic clam *Macoma balthica* in Chesapeake Bay. Marine Ecology Progress Series. 2003;257:179–88. 10.3354/meps257179

[36] Stickle WB, Kapper MA, Liu L-L, Gnaiger E, Wang SY. Metabolic adaptations of several species of crustaceans and molluscs to hypoxia: tolerance and microcalorimetric studies. The Biological Bulletin. 1989;177(2):303–12. 10.2307/1541945

[37] Alexander JE Jr, McMahon RF. Respiratory response to temperature and hypoxia in the zebra mussel *Dreissena polymorpha*. Comparative Biochemistry and Physiology Part A: Molecular & Integrative Physiology. 2004;137(2):425–34. 10.1016/j.cbpb.2003.11.003 15123216

[38] Nie H, Chen P, Huo Z, Chen Y, Hou X, Yang F, et al Effects of temperature and salinity on oxygen consumption and ammonia excretion in different colour strains of the Manila clam, *Ruditapes philippinarum*. Aquaculture Research. 2017;48(6):2778–86. 10.1111/are.13111

[39] Le Moullac G, Quéau I, Le Souchu P, Pouvreau S, Moal J, René Le Coz J, et al Metabolic adjustments in the oyster *Crassostrea gigas* according to oxygen level and temperature. Marine Biology Research. 2007;3(5):357–66. 10.1080/17451000701635128

[40] Liu C, Shin P, Cheung S. Comparisons of the metabolic responses of two subtidal nassariid gastropods to hypoxia and re-oxygenation. Marine Pollution Bulletin. 2014;82(1–2):109–16. 10.1016/j.marpolbul.2014.03.013 24680715

[41] Venter L, Mienie LJ, van Rensburg PJJ, Mason S, Vosloo A, Lindeque JZ. Uncovering the metabolic response of abalone (Haliotis midae) to environmental hypoxia through metabolomics. Metabolomics. 2018;14(4):49 10.1007/s11306-018-1346-8 30830330

[42] Gray JS, Wu RS-s, Or YY. Effects of hypoxia and organic enrichment on the coastal marine environment. Marine Ecology Progress Series. 2002;238:249–79. 10.3354/meps238249

[43] Lushchak V, Bahnjukova T, Storey K. Effect of hypoxia on the activity and binding of glycolytic and associated enzymes in sea scorpion tissues. Brazilian Journal of Medical and Biological Research. 1998;31(8):1059–67. 10.1590/S0100-879X1998000800005 9777012

[44] Mahfouz ME, Hegazi MM, El-Magd MA, Kasem EA. Metabolic and molecular responses in Nile tilapia, Oreochromis niloticus during short and prolonged hypoxia. Marine and Freshwater Behavior and Physiology. 2015;48(5):319–40. 10.1080/10236244.2015.1055915

[45] Anestis A, Pörtner HO, Michaelidis B. Anaerobic metabolic patterns related to stress responses in hypoxia exposed mussels *Mytilus galloprovincialis*. Journal of Experimental Marine Biology and Ecology. 2010;394(1–2):123–33. 10.1016/j.jembe.2010.08.008

[46] Sussarellu R, Fabioux C, Sanchez MC, Le Goïc N, Lambert C, Soudant P, et al Molecular and cellular response to short-term oxygen variations in the Pacific oyster *Crassostrea gigas*. Journal of Experimental Marine Biology and Ecology. 2012;412:87–95. 10.1016/j.jembe.2011.11.007

[47] Bumett LE, Stickle WB. Physiological responses to hypoxia. Coastal Hypoxia: Consequences for Living Resources and Ecosystems. 2001;58:101–14.

[48] Zwaan A, Dando P. Phosphoenolpyruvate-pyruvate metabolism in bivalve molluscs. Molecular Physiology. 1984;5:285–312.

[49] Harcet M, Perina D, Pleše B. Opine dehydrogenases in marine invertebrates. Biochemical Genetics. 2013;51(9–10):666–76. 10.1007/s10528-013-9596-7 23644944

[50] Sato M, Takahara M, Kanno N, Sato Y, Ellington WR. Isolation of a new opine, β-alanopine, from the extracts of the muscle of the marine bivalve mollusc, *Scapharca broughtonii*. Comparative Biochemistry and Physiology Part B: Comparative Biochemistry. 1987;88(3):803–6. 10.1016/0305-0491(87)90247-1

[51] Ying Z, Huifeng W, Lei W, Zeping X, Bo G. Effects of hypoxia in the gills of the Manila clam *Ruditapes philippinarum* using NMR-based metabolomics. Marine Pollution Bulletin. 2017;114:84–89 10.1016/j.marpolbul.2016.08.066 27587234

[52] Donaghy L, Artigaud S, Sussarellu R, Lambert C, Le Goïc N, Hégaret H, et al Tolerance of bivalve mollusc hemocytes to variable oxygen availability: a mitochondrial origin? Aquatic Living Resources. 2013;26(3):257–61. 10.1051/alr/2013054

[53] Ivanina AV, Nesmelova I, Leamy L, Sokolov EP, Sokolova IM. Intermittent hypoxia leads to functional reorganization of mitochondria and affects cellular bioenergetics in marine molluscs. Journal of Experimental Biology. 2016;219(11):1659–74. 10.1242/jeb.134700 27252455

[54] Kurochkin IO, Ivanina AV, Eilers S, Downs CA, May LA, Sokolova IM. Cadmium affects metabolic responses to prolonged anoxia and reoxygenation in eastern oysters (Crassostrea virginica). American Journal of Physiology-Regulatory, Integrative and Comparative Physiology. 2009;297(5):R1262–R72. 10.1152/ajpregu.00324.2009 19726715

[55] Honda HM, Korge P, Weiss JN. Mitochondria and ischemia/reperfusion injury. Annals of the New York Academy of Sciences. 2005;1047(1):248–58. 10.1196/annals.1341.022 16093501

[56] Laudien J, Schiedek D, Brey T, Pörtner H-O, Arntz W. Survivorship of juvenile surf clams Donax serra (Bivalvia, Donacidae) exposed to severe hypoxia and hydrogen sulphide. Journal of Experimental Marine Biology and Ecology. 2002;271(1):9–23. 10.1016/S0022-0981(02)00030-8

[57] Nilsson HC, Rosenberg R. Hypoxic response of two marine benthic communities. Marine Ecology Progress Series. 1994;115(3):209–17.

[58] Haselmair A, Stachowitsch M, Zuschin M, Riedel B. Behaviour and mortality of benthic crustaceans in response to experimentally induced hypoxia and anoxia in situ. Marine Ecology Progress Series. 2010;414:195–208. 10.3354/meps08657

[59] Riedel B, Zuschin M, Stachowitsch M. Tolerance of benthic macrofauna to hypoxia and anoxia in shallow coastal seas: a realistic scenario. Marine Ecology Progress Series. 2012;458:39–52. 10.3354/meps09724

