# Supplementary material for: Effects of hypoxia on survival, behavior, metabolism and cellular damage of Manila clam (Ruditapes philippinarum)
Source: PLoS One. 2019 Apr 18;14(4):e0215158. doi: 10.1371/journal.pone.0215158 (PMC6472746; doi:10.1371/journal.pone.0215158)
Supplement: S1 Table — (DOCX) [file pone.0215158.s001.docx]

|  | Normality | Homogeneity | Method | Statistic | Significance |
| --- | --- | --- | --- | --- | --- |
| Survival |  |  | Kruskal–Wallis H | 10.692 | 0.014 |
| Burial rates |  |  | Kruskal–Wallis H | 10.645 | 0.014 |
| OCR (day 10) | Yes | No | Kruskal–Wallis H | 12.707 | < 0.001 |
| OCR (day 20) | Yes | No | Kruskal–Wallis H | 9.620 | 0.022 |
| AER (day 10) | Yes | No | Kruskal–Wallis H | 20.707 | < 0.001 |
| AER (day 20) | Yes | Yes | ANOVA | 46.821 | < 0.001 |
| O : N (day 10) | Yes | No | Kruskal–Wallis H | 14.082 | < 0.001 |
| O : N (day 20) | Yes | No | Kruskal-Wallis H | 13.204 | < 0.001 |
| LDH (day 10) | Yes | Yes | ANOVA | 1.125 | 0.356 |
| LDH (day 20) | Yes | No | Kruskal–Wallis H | 16.416 | 0.001 |
| PFK (day 10) | Yes | Yes | ANOVA | 5.163 | < 0.001 |
| PFK (day 20) | Yes | Yes | ANOVA | 3.081 | 0.045 |
| PK (day 10) | Yes | Yes | ANOVA | 4.134 | 0.015 |
| PK (day 20) | Yes | Yes | ANOVA | 3.235 | 0.038 |
