# Supplementary material for: Effects of hypoxia on survival, behavior, metabolism and cellular damage of Manila clam (Ruditapes philippinarum)
Source: PLoS One. 2019 Apr 18;14(4):e0215158. doi: 10.1371/journal.pone.0215158 (PMC6472746; doi:10.1371/journal.pone.0215158)
Supplement: S2 Table — (DOCX) [file pone.0215158.s002.docx]

**Measures of dissolved oxygen concentration levels in Experiment 1**

| Day | Dissolved oxygen (mg L^-1^) | | | |
| --- | --- | --- | --- | --- |
| 1 | 5.98 | 2.00 | 1.01 | 0.52 |
| 2 | 5.96 | 1.97 | 1.00 | 0.51 |
| 3 | 5.95 | 1.99 | 1.02 | 0.50 |
| 4 | 5.99 | 2.04 | 0.97 | 0.50 |
| 5 | 5.95 | 2.03 | 1.03 | 0.51 |
| 6 | 6.00 | 1.97 | 0.97 | 0.52 |
| 7 | 5.99 | 1.98 | 1.00 | 0.51 |
| 8 | 6.02 | 2.05 | 1.01 | 0.51 |
| 9 | 6.01 | 1.99 | 1.05 | 0.50 |
| 10 | 5.97 | 2.03 | 0.96 | 0.49 |
| 11 | 6.07 | 2.05 | 1.00 | 0.50 |
| 12 | 6.03 | 2.04 | 1.03 | 0.52 |
| 13 | 6.00 | 1.97 | 0.98 | 0.51 |
| 14 | 6.01 | 2.05 | 1.01 | 0.49 |
| 15 | 6.06 | 1.97 | 1.00 | 0.52 |
| 16 | 6.07 | 2.04 | 1.02 | 0.47 |
| 17 | 5.99 | 2.00 | 0.96 | 0.53 |
| 18 | 6.02 | 1.99 | 0.96 | 0.52 |
| 19 | 5.96 | 2.03 | 1.00 | 0.49 |
| 20 | 6.02 | 2.00 | 1.02 | 0.54 |

**Measures of dissolved oxygen concentration levels in Experiment 2.1**

| Day | Dissolved oxygen (mg L^-1^) | | | |
| --- | --- | --- | --- | --- |
| 1 | 6.02 | 0.80 | 0.60 | 0.41 |
| 2 | 6.00 | 0.79 | 0.60 | 0.41 |
| 3 | 5.99 | 0.80 | 0.59 | 0.39 |
| 4 | 6.01 | 0.80 | 0.59 | 0.41 |
| 5 | 5.96 | 0.79 | 0.60 | 0.40 |
| 6 | 5.98 | 0.81 | 0.60 | 0.39 |
| 7 | 6.01 | 0.80 | 0.61 | 0.42 |
| 8 | 5.98 | 0.79 | 0.60 | 0.41 |
| 9 | 6.02 | 0.80 | 0.59 | 0.40 |
| 10 | 6.04 | 0.79 | 0.59 | 0.40 |
| 11 | 6.06 | 0.80 | 0.59 | 0.40 |
| 12 | 6.00 | 0.80 | 0.61 | 0.40 |
| 13 | 6.05 | 0.81 | 0.60 | 0.39 |
| 14 | 6.02 | 0.79 | 0.61 | 0.40 |
| 15 | 6.00 | 0.81 | 0.60 | 0.41 |
| 16 | 5.98 | 0.80 | 0.61 | 0.40 |
| 17 | 5.98 | 0.80 | 0.61 | 0.40 |
| 18 | 6.00 | 0.80 | 0.60 | 0.40 |
| 19 | 6.01 | 0.80 | 0.59 | 0.40 |
| 20 | 5.97 | 0.80 | 0.60 | 0.40 |

**Measures of dissolved oxygen concentration levels in Experiment 2.2**

| Day | Dissolved oxygen (mg L^-1^) | | | |
| --- | --- | --- | --- | --- |
| 1 | 6.05 | 0.86 | 0.65 | 0.46 |
| 2 | 6.00 | 0.86 | 0.66 | 0.45 |
| 3 | 5.95 | 0.87 | 0.64 | 0.44 |
| 4 | 5.93 | 0.87 | 0.65 | 0.47 |
| 5 | 6.11 | 0.85 | 0.65 | 0.46 |
| 6 | 5.95 | 0.86 | 0.64 | 0.46 |
| 7 | 6.04 | 0.84 | 0.64 | 0.46 |
| 8 | 6.06 | 0.86 | 0.67 | 0.47 |
| 9 | 6.02 | 0.86 | 0.66 | 0.45 |
| 10 | 6.03 | 0.84 | 0.63 | 0.45 |
| 11 | 5.94 | 0.84 | 0.66 | 0.44 |
| 12 | 6.00 | 0.85 | 0.66 | 0.45 |
| 13 | 5.89 | 0.86 | 0.66 | 0.46 |
| 14 | 5.96 | 0.87 | 0.66 | 0.46 |
| 15 | 6.08 | 0.85 | 0.65 | 0.46 |
| 16 | 6.06 | 0.84 | 0.67 | 0.46 |
| 17 | 5.99 | 0.85 | 0.64 | 0.44 |
| 18 | 6.01 | 0.85 | 0.65 | 0.44 |
| 19 | 6.09 | 0.86 | 0.66 | 0.46 |
| 20 | 6.00 | 0.85 | 0.64 | 0.47 |

**Measures of dissolved oxygen concentration levels in Experiment 2.3**

| Day | Dissolved oxygen (mg L^-1^) | | | |
| --- | --- | --- | --- | --- |
| 1 | 6.03 | 0.92 | 0.72 | 0.49 |
| 2 | 6.01 | 0.90 | 0.70 | 0.51 |
| 3 | 5.97 | 0.91 | 0.68 | 0.50 |
| 4 | 6.00 | 0.93 | 0.69 | 0.48 |
| 5 | 5.95 | 0.91 | 0.70 | 0.50 |
| 6 | 5.99 | 0.93 | 0.71 | 0.49 |
| 7 | 5.99 | 0.90 | 0.70 | 0.52 |
| 8 | 6.01 | 0.92 | 0.70 | 0.50 |
| 9 | 6.03 | 0.90 | 0.71 | 0.52 |
| 10 | 6.04 | 0.89 | 0.68 | 0.49 |
| 11 | 6.04 | 0.89 | 0.72 | 0.51 |
| 12 | 5.99 | 0.92 | 0.71 | 0.50 |
| 13 | 6.02 | 0.90 | 0.72 | 0.50 |
| 14 | 6.01 | 0.90 | 0.71 | 0.47 |
| 15 | 6.05 | 0.89 | 0.69 | 0.50 |
| 16 | 6.01 | 0.92 | 0.70 | 0.49 |
| 17 | 5.98 | 0.91 | 0.71 | 0.53 |
| 18 | 6.01 | 0.90 | 0.71 | 0.50 |
| 19 | 6.01 | 0.90 | 0.71 | 0.52 |
| 20 | 5.96 | 0.92 | 0.70 | 0.50 |

**Measures of dissolved oxygen concentration levels in Experiment 2.4**

| Day | Dissolved oxygen (mg L^-1^) | | | |
| --- | --- | --- | --- | --- |
| 1 | 6.01 | 0.95 | 0.77 | 0.55 |
| 2 | 6.08 | 0.95 | 0.77 | 0.55 |
| 3 | 5.95 | 0.96 | 0.75 | 0.57 |
| 4 | 6.12 | 0.94 | 0.76 | 0.56 |
| 5 | 6.08 | 0.94 | 0.77 | 0.53 |
| 6 | 5.94 | 0.95 | 0.73 | 0.56 |
| 7 | 5.92 | 0.96 | 0.75 | 0.55 |
| 8 | 6.00 | 0.94 | 0.75 | 0.54 |
| 9 | 6.06 | 0.95 | 0.76 | 0.55 |
| 10 | 6.04 | 0.96 | 0.75 | 0.54 |
| 11 | 6.09 | 0.94 | 0.73 | 0.54 |
| 12 | 6.07 | 0.94 | 0.75 | 0.54 |
| 13 | 5.95 | 0.95 | 0.74 | 0.56 |
| 14 | 6.04 | 0.94 | 0.74 | 0.56 |
| 15 | 6.09 | 0.96 | 0.77 | 0.54 |
| 16 | 6.11 | 0.95 | 0.74 | 0.56 |
| 17 | 5.94 | 0.95 | 0.75 | 0.57 |
| 18 | 6.05 | 0.94 | 0.75 | 0.55 |
| 19 | 6.10 | 0.96 | 0.75 | 0.55 |
| 20 | 6.00 | 0.94 | 0.75 | 0.55 |
